# Physiological Roles of DNA Double-Strand Breaks

**DOI:** 10.1155/2017/6439169

**Published:** 2017-10-18

**Authors:** Farhaan A. Khan, Syed O. Ali

**Affiliations:** School of Clinical Medicine, Addenbrooke's Hospital, University of Cambridge, Hills Road, Cambridge CB2 0SP, UK

## Abstract

Genomic integrity is constantly threatened by sources of DNA damage, internal and external alike. Among the most cytotoxic lesions is the DNA double-strand break (DSB) which arises from the cleavage of both strands of the double helix. Cells boast a considerable set of defences to both prevent and repair these breaks and drugs which derail these processes represent an important category of anticancer therapeutics. And yet, bizarrely, cells deploy this very machinery for the intentional and calculated disruption of genomic integrity, harnessing potentially destructive DSBs in delicate genetic transactions. Under tight spatiotemporal regulation, DSBs serve as a tool for genetic modification, widely used across cellular biology to generate diverse functionalities, ranging from the fundamental upkeep of DNA replication, transcription, and the chromatin landscape to the diversification of immunity and the germline. Growing evidence points to a role of aberrant DSB physiology in human disease and an understanding of these processes may both inform the design of new therapeutic strategies and reduce off-target effects of existing drugs. Here, we review the wide-ranging roles of physiological DSBs and the emerging network of their multilateral regulation to consider how the cell is able to harness DNA breaks as a critical biochemical tool.

## 1. Introduction

Just as DNA breakage can devastate genomic integrity, it can also be deliberately and precisely exploited by cells in feats of genetic craftsmanship. Indeed, various integral cellular processes and genetic transactions are underpinned by the measured use of such potentially deleterious breaks.

The genome is continuously exposed to a panoply of DNA damaging agents, both endogenous and exogenous, which pose a considerable threat to genomic integrity. These genotoxic insults result in diverse DNA lesions including mismatches, base adducts, pyrimidine dimers, intra- and interstrand cross links, DNA-protein adducts, and strand breaks [1]. Among the most noxious lesions are DNA double-strand breaks (DSBs) wherein both strands of the double helix are broken through cleavage of phosphodiester linkages in the backbone of the duplex [2]. There is great variation among DSBs both structurally and in terms of the mechanisms of their generation. While simple DSBs such as those generated by restriction endonucleases may have either blunt or staggered ends, DSBs induced by physical or chemical agents, such as by ionising radiation and radiomimetic chemicals, both of which are used in cancer therapy, can display increased complexity. This includes chemical modifications of termini, differing latency of generation, indirect DSB formation from processing of other forms of DNA damage, and chromatin destabilisation from clustering of DSBs [3].

DSBs are highly mutagenic and can induce potentially tumorigenic chromosomal translocations. If unrepaired, DSBs may also lead to cell death [4]. It is thus of utmost primacy that such cytotoxic breaks are rapidly detected, signalled, and repaired. Indeed, DSBs elicit a potent DNA damage response (DDR) comprising DNA repair, cell cycle arrest, and/or apoptosis [2]. Two major pathways operate in eukaryotic cells in the repair of endogenously or exogenously induced DSBs: nonhomologous end-joining (NHEJ) and homologous recombination (HR). Unlike error-prone NHEJ which operates throughout the cell cycle, HR is essentially error-free and is limited to the S and G2 phases [5].

Despite the dangers inherent in such lesions, DSBs are precisely employed for the intentional but controlled disruption of genomic integrity in biological processes. The cellular roles of physiological DSBs can be broadly considered as one of two major functionalities: genetic recombination or manipulation of DNA topology. In the former role, DSBs can act as genomic “shufflers,” carefully but permanently recombining DNA segments for genomic diversification in lymphocytes [6, 7] and germ cells [8]. In contrast, topoisomerase-mediated DSBs serve as genomic “sculptors,” modulating higher-order DNA structure and serving to facilitate DNA replication and transcription [9–11], regulate gene expression [12–15], and alter chromatin state [16, 17]. While this latter form of DSB may, at first glance, appear to be little more than a transient intermediate, these “cleavage complexes” are structurally DSBs, also exist as longer-lived species, can be endogenously or exogenously converted into abortive breaks, and are highly spatiotemporally regulated to both produce functionally diverse genomic contortions and prevent genotoxicity or failure of genetic transactions [18, 19]. Hence, both sources of DSB will be considered in this discussion.

**Figure 1:**
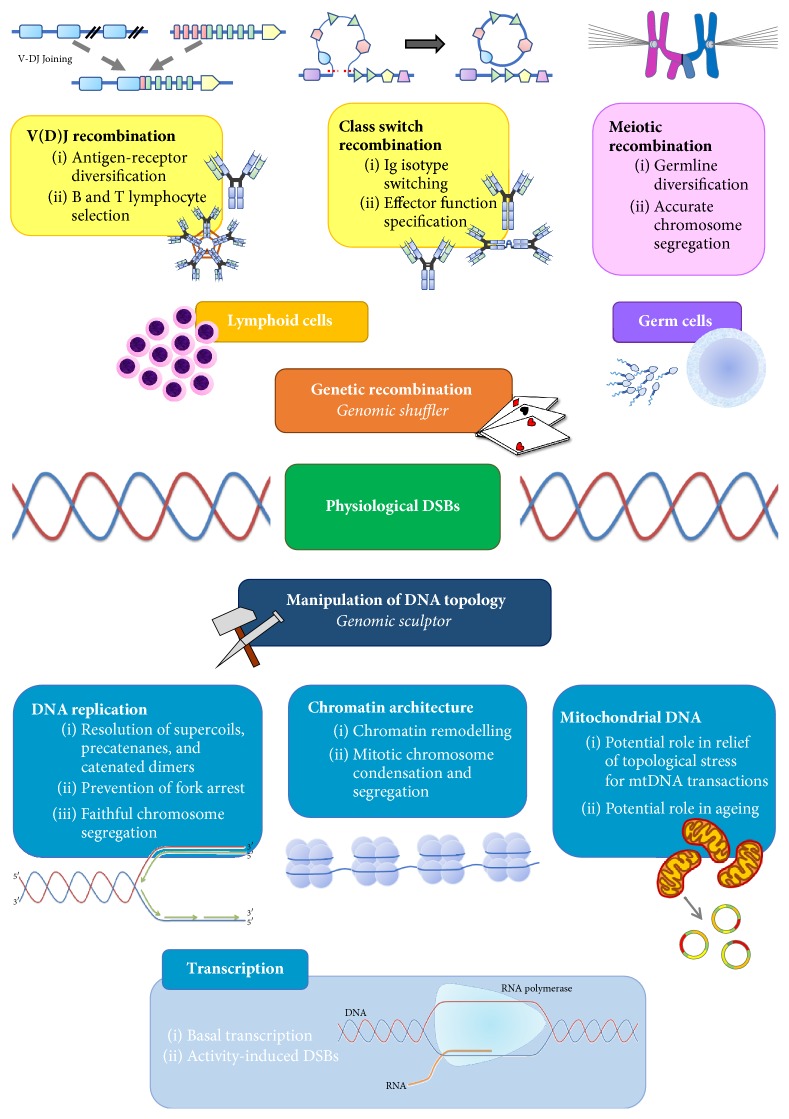
Summary of the diverse roles of physiological DSBs in biological processes.

Here, we review the wide-ranging biological functions of these various physiological DSBs and the multilayered regulation thereof, together constituting cellular “DSB physiology” (Figure 1). By considering DSB physiology in its various forms, we explore how the cell is able to harness potentially destructive DSBs in delicate genetic operations.

## 2. Genetic Recombination: DSBs as Genomic Shufflers

### 2.1. Physiological DSBs in V(D)J Recombination

The role of physiological DSBs in the diversification of the adaptive immune response is well documented. V(D)J recombination describes the process whereby lymphoid cells recombine a repertoire of germline variable (V), diversity (D), and joining (J) exon gene segments in various permutations to generate enormous antigen-receptor diversity in immunoglobulins (Ig) and T cell receptors (TCRs). All three exon segments are used for assembling the variable region of the Ig heavy chain and the TCR *β* and *δ* chains, whereas only V and J segments are required for the Ig light chain and TCR *α* and *γ* chains [20]. The process involves large genomic rearrangements and DSBs act in these processes as “recombinogenic biomarkers,” specifying the recruitment of the recombinatorial machinery. It thus follows that the spatiotemporal regulation of DSB induction and repair is critical to the precise execution of this genetic shuffling, and perturbations thereof can produce disease in humans. The mechanism proceeds as two broad phases, cleavage and joining, corresponding to the processes of DSB formation and DSB resolution, respectively.

#### 2.1.1. Cleavage: DSB Formation

Each V, D, and J segment is flanked by a recombination signal sequence (RSS), comprising conserved heptamer-spacer-nonamer sequence elements [21, 22]. Cleavage is initiated by the recognition of these RSS elements by the lymphoid-cell specific recombinase complex [23, 24]. Components of this complex, RAG-1 and RAG-2, bind one RSS from each of the paired gene segments, to form RAG-RSS complexes associated with each signal sequence [25, 26]. The two RSS groups are then approximated and synapsis proceeds to form a precleavage synaptic complex (PcSC). The PcSC is typically directed between gene segments with different RSS spacer lengths (either 12 or 23 base pairs) conforming to the 12/23 restriction rule [27]. PcSC formation is facilitated by the DNA-bending high-mobility group proteins HMGB1 and HMGB2 which are widely involved in chromatin architecture [28]. Here, HMGB1/2 act to enhance RAG1/2 binding to and cleavage of the 23 RSS, possibly via stabilisation of RAG-mediated bending of the 23 RSS spacer [29–33]. The RAG proteins proceed to introduce a nick at the junction between the RSS heptamer and coding sequence. While RAG-1 alone but not RAG-2 possesses DNA-binding activity and can mediate signal recognition, association of RAG-1 with RAG-2 increases sequence specificity and the stability of the enzyme-DNA complex [34, 35]. Nick formation is followed by a RAG1-mediated transesterification reaction forming a DSB comprising a blunt 5′-phosphorylated signal end and a hairpin coding end [23, 36]. The DSB is then enclosed in a postcleavage synaptic complex before NHEJ repair in the joining phase [37].

#### 2.1.2. Joining: DSB Resolution

Prior to joining, hairpin coding ends are nicked open through the endonuclease activity of the nuclear enzyme Artemis, which complexes with the serine/threonine kinase DNA-PKcs, which, in turn, activates Artemis by phosphorylation [38, 39]. However, studies also indicate a postcleavage role for RAG proteins in opening hairpin coding ends through 3′-flap endonuclease activity [40–42] as well as serving as a scaffold to shield the DNA ends from aberrant nuclease digestion [43]. Modifications during recombination are introduced through Artemis-mediated asymmetric hairpin opening and/or terminal deoxynucleotidyl transferase- (TdT-) mediated nucleotide addition. The former mechanism enables palindromic repeat or “P” nucleotide addition as well as nucleotide loss from coding ends [44], while TdT-mediated addition inserts random “N” nucleotides at coding ends in a 5′-to-3′ direction [38, 45]. TdT, a template-independent polymerase, is recruited by a complex of NHEJ repair proteins comprising XRCC4, DNA ligase IV, and DNA-PK (itself a complex of Ku70, Ku86, and DNA-PKcs) [46–49]. The subsequent activity of DNA polymerase *λ* and *μ* combined with exonucleases generates compatible coding ends [50, 51] which are then ligated by the XRCC4-DNA ligase IV-XLF complex to produce the recombined V(D)J product [46, 47, 52].

#### 2.1.3. Regulation of DSBs in V(D)J Recombination

An important point of control in the DSB formation step of V(D)J recombination is the cell cycle-dependent regulation of RAG expression. Cyclin A-CDK2 phosphorylation-mediated degradation of RAG-2 at the G1/S transition [53] exerts temporal control over RAG-induced DSBs by restricting RAG2 expression to the G1 phase of the cell cycle [54]. Furthermore, in p53-deficient mice a nonphosphorylatable T490A mutation of the phosphodegron in the C-terminus of RAG-2 results in increased lymphoma formation characterised by an increased frequency of clonal chromosomal translocations [55], illustrating the importance of this regulation for the integrity of recombination.

Additional temporal control is exerted over RSS selection for DSB formation and only certain RSS sites are available for recombination, exhibiting cell- and stage-specific biases [56, 57]. The great accuracy of the recombinatorial machinery, both in RSS specification and in avoiding inauthentic RSS-like (cryptic RSS; cRSS) sites, which occur around once per 600 bases in random DNA sequences [58], involves controlling the accessibility of the recombinase to its substrate for DSB generation. The discovery of the concurrence of IgH variable region (V_H_) germline transcription with its joining with DJ_H_ suggested an open chromatin state was required for recombination [57]. This “accessibility hypothesis” is now thought to involve chromatin architectural changes brought about by DNA and epigenetic modifications, chromatin-binding proteins, and cis-acting enhancer elements. These ultimately produce a transcriptionally active locus characterised by DNA hypomethylation, activating histone modifications, DNase I sensitivity, and increased RNA polymerase II occupancy [59–64]. RAG2 has been shown to specifically recognise the histone modification H3K4me3, a marker enriched at active promoters, via its PHD finger, an interaction which also stimulates RAG complex activity [65, 66]. This recognition likely serves to localise the recombinase to target RSSs by active promoters and upregulate recombination thereat [64]. Modulation of chromatin packing also determines the order of rearrangement observed in B cell Ig (heavy chain before light chain) and T cell TCR (*β* chain before *α* chain), as well as the process of allelic exclusion [56]. Aside from modulation of accessibility of RSS sites to binding and cleavage by the recombination machinery, target locus position within chromatin compartments of the nucleus and the structure of the locus itself are also directed to facilitate recombination [64].

#### 2.1.4. Dysregulation of DSB Physiology in V(D)J Recombination

Broadly, perturbations in V(D)J recombination can be from either an inability to form DSBs, an inability to repair them, or inaccurate pairing of RSSs. Although viable, Rag-1- or Rag-2-deficient mice are unable to carry out V(D)J recombination and lack mature B and T lymphocytes, presenting with severe combined immune deficiency (SCID) [67, 68]. Missense mutations in either* RAG-1* or* RAG-2* genes are also observed in humans with an analogous form of autosomal recessive SCID known as Omenn syndrome [69]. Genetic studies of polymorphisms in wild-type and Omenn syndrome patients as well as biochemical data characterising recombinatorial competence of Omenn syndrome mutants suggest that* RAG-1* and* RAG-2* mutants exhibit lower efficiency of V(D)J recombination [69]. These mutations are associated with decreased RAG DNA-binding and cleavage activity and a lower efficiency of interaction between RAG proteins [69].

Impairment of the joining phase of V(D)J recombination can also cause immunodeficiency. While all mammalian NHEJ pathway mutants display sensitivity to ionising radiation and a lack of B and T lymphocytes, a spectrum of phenotypes is observed in mouse models. DNA-PKcs and Artemis mutants exhibit subtle defects in DSB repair [70] but display no growth retardation [71]. Hairpin opening is lost in Artemis-deficient SCID [72] and* DCLRE1C* hypomorphism in humans has been found to underlie an Omenn syndrome phenotype associated with defective endonuclease activity [73]. Ku70 and Ku80 mutants display similar phenotypes of radiosensitivity and growth retardation [74, 75] likely due to the interdependence conferred by their heterodimerisation [76]. Mutants of the critical end-joining proteins XRCC4 and DNA ligase IV have the most severe phenotype, both displaying V(D)J recombination defects, impaired lymphocyte development, p53-dependent neuronal apoptosis, and embryonic lethality [77–79]. These findings thus point towards a crucial role of DSB repair in B/T cell physiology and adaptive immune diversification.

Lymphomas have been found to exhibit a range of chromosomal aberrations as a result of erroneous DSB handling in V(D)J recombination. Oncogenic lesions have been found to include chromosomal translocations, insertions, deletions, and inversions and broadly result from errors in RSS pairing or joining [80–82]. Inaccurate RSS recognition can produce RSS/cRSS pairings which may exert tumorigenic effects via amplification of a protooncogene or juxtaposition of transcriptional regulatory elements from the antigen-receptor locus with a protooncogene, driving dysregulated expression [6]. cRSS/cRSS pairings may also arise which can produce translocations or deletions [6]. Aberrant DSB formation has also been found to underlie the reciprocal (t14; 18) translocation found in 90% of follicular lymphomas [83, 84]. In this case, it has been shown that cleavage of non-B-form DNA at a non-RSS “translocation fragile zone” called the major breakpoint region (MBR) at the Bcl-2 gene produces a translocation with the DSB at the Ig heavy chain locus on chromosome 14 [84]. Recent work on minichromosomal substrates has revealed the importance of CpG methylation in the localisation of aberrant DSB formation at the Bcl-2 MBR and that breakage is AID-dependent (activation-induced cytidine deaminase) [85]. Lymphomas have also been found to exhibit defects in DSB joining producing chromosome translocations by the “end donation” model. This involves the erroneous joining of a RAG-induced DSB at an authentic RSS with a non-RAG-mediated DSB [86].

### 2.2. Physiological DSBs in Class Switch Recombination

Class switch recombination (CSR) is another example of a DSB-dependent recombination event, occurring in mature B cells. In response to antigenic stimuli and costimulatory signals from other immune cells, B cells undergo CSR to alter their Ig constant heavy chain while retaining the same antigen specificity, in effect, generating an alternative Ig isotype with the same paratope but distinct effector functions [87]. This enables a permanent switch of expression from the default IgM and IgD to alternative Ig isotypes (IgA, IgG, or IgE) by recombination of the heavy chain (IgH) locus. Recombination is achieved through cleavage and excision of constant heavy chain (C_H_) genes via DSB generation, followed by ligation of the remaining segments through DSB resolution [87].

Conserved motifs called switch (S) regions upstream of each C_H_ gene are the site of DSB generation [88, 89]. AID initiates recombination by conversion of multiple S region deoxycytidine residues into deoxyuracil [90–93]. The resulting UG mismatch can be converted into a DSB through base-excision repair (BER) and/or mismatch repair (MMR) DNA repair pathways [87]. BER proceeds through excision of deoxyuracil by uracil DNA glycosylase (UNG) [93, 94] followed by nicking of the resulting abasic site by apurinic/apyrimidinic endonucleases (APE) [87, 95]. AID-mediated deamination on the opposite strand introduces a second nick in close proximity, thereby generating a staggered DSB [89, 96]. Processing of these overhangs forms blunt free DNA ends which are permissive for shuffling [87, 97].

As aforementioned, MMR is another pathway employed in CSR DSB generation and is thought to provide auxiliary support to BER-mediated mismatch excision [98]. Indeed, multiple studies of MMR-deficient murine B cells have shown impaired and aberrant CSR implicating MMR proteins Msh2, Msh6, Mlh1, Pms2, and Exo1 in the recombination process [99–104]. One model proposes that while the aforementioned BER-induced SSBs can automatically form DSBs if near one another, distant SSBs within the large S regions can be converted into DSBs by assistance from MMR [105]. This first involves recognition of an AID-induced UG mismatch by a heterodimer of Msh2-Msh6, followed by recruitment of the endonuclease heterodimer Mlh1-Pms2. These proteins, in turn, recruit the 5′-3′ exonuclease Exo1 to a nearby BER-induced 5′ SSB and Exo1 proceeds to excise the strand towards the mismatch and beyond until it reaches another SSB, thus producing a DSB [105]. Alternatively it has also been proposed that if Exo1-generated single-stranded DNA gaps are formed on opposite strands, this too may form a DSB [106].

After intrachromosomal deletion of the intervening unwanted C_H_ genes, the free end of the variable domain region is ligated to that of the new constant domain exon by the NHEJ machinery [107–110].

#### 2.2.1. Regulation of DSBs in CSR

Recombination underlying isotype switching of constant heavy chain genes is regulated by germline transcripts which modify switch region accessibility to the DNA handling machinery [105]. Transcription beginning at germline promoters, upstream of a target S region, generates a sterile (noncoding) transcript known as a germline transcript (GT) [111, 112]. Activation of specific cytokine-inducible transcription factors and their action at germline promoters determines which GT is generated [113]. These germline transcripts direct AID to specific S regions thus spatially regulating DSB generation and, in turn, CSR [105, 114].

AID deamination can only occur on single-stranded DNA (ssDNA) [115] and one proposed mechanism of GT-mediated regulation is optimisation of S region structure for AID recognition [115]. This could be achieved through the creation of short ssDNA tracts in the S regions, paralleling somatic hypermutation [116], or through RNA-DNA hybrid R-loop formation [117, 118]. AID can also be directly recruited to RNA Pol II at the initiation or elongation phase of GT formation, which may facilitate AID targeting [105, 119]. Further regulation may also occur at the level of higher-order chromatin structure whereby histone modifications in the transcribed region may modulate AID accessibility [120]. While the exact mechanism of this architectural control is unclear, it appears to be associated with B cell activation, AID expression, and RNA Pol II enrichment [120, 121]. It is thus clear that various levels of regulation act to spatiotemporally restrain DSB formation and repair in CSR.

### 2.3. Physiological DSBs in Meiotic Recombination

Beyond its roles in lymphoid cells, DSB-induced recombination also acts in meiosis where it is essential for both the fidelity of chromosome segregation and diversification of the germline. Meiosis is a form of reductive cell division exclusive to gametogenesis and involves two successive divisions following replication, known as MI and MII [122]. A hallmark of meiosis is meiotic recombination between homologous chromosome pairs or dyads, which is reliant on DSB induction and HR repair, resulting in exchange of genetic information between nonsister chromatids [122]. These “crossover” events essentially produce shuffling of alleles in each chromatid, giving rise to a unique haploid complement in the resultant gametes, ultimately creating genetic diversity within the population. Furthermore, meiotic recombination establishes physical linkages between homologs allowing accurate segregation by the spindle apparatus [123]. Just as in V(D)J recombination and CSR, careful spatiotemporal regulation of DNA break induction and resolution is critical to the integrity of the process.

#### 2.3.1. DSB Formation

Meiotic recombination is initiated by DSB induction by a dimer of the topoisomerase-like transesterase Spo11 which acts via a covalently linked protein tyrosyl-DNA intermediate complex [124–127]. Interestingly, the human SPO11 homolog maps to position 20q13.2-q13.3, a region known to be amplified in some breast and ovarian tumours, possibly reflecting genomic instability due to dysregulation of DSB formation [128]. Spo11 action is critical for gametogenesis and loss in mice produces infertility and results in defective meiotic recombination, DSB formation, and synapsis in* Spo11*^−/−^ mouse spermatocytes [129]. DSB formation is also dependent on an extensive protein-protein interaction network, most studied in* S. cerevisiae*, between Spo11 and other meiotic proteins. These proteins include components of the Mre11-Rad50-Xrs2 (MRX) complex and multiple Spo11-accessory proteins including Mei4, Mer2, Rec102, Rec104, Rec114, and Ski8 which facilitate DSB formation [126, 130]. The functions of these various proteins are incompletely understood; however, the WD repeat protein Ski8 has been found to stabilise association of Spo11 on meiotic chromatin and promotes recruitment of other accessory proteins [130] and Rec102 and Rec104 are known to form a multiprotein complex with Spo11 and act with Rec114 to regulate Spo11 self-association [131–133]. Rec 114, Mer2, and Mei4 (RMM) constitute a separate subcomplex [134] which plays a role in the spatiotemporal regulation of DSB formation as will be discussed below.

#### 2.3.2. DSB Resolution

The resulting DSBs then undergo endonucleolytic processing which releases Spo11 to allow repair [135]. This initial DSB resection is coordinated with cell cycle progression and is dependent on the activity of the multisubunit nuclease MRX complex and Sae2 protein in budding yeast [126, 136–138]. The former complex also acts to facilitate HR independently of its nucleolytic activity [139]. Analogous to the MRX complex, the MRE11-RAD50-NBS1 (MRN) complex possesses a similar role in SPO11 removal in other eukaryotes [140]. Spo11 removal is followed by extension of the end resection, involving the helicase Sgs1 and nucleases Exo1 and Dna2, forming 3′ single-stranded overhangs for HR [138, 141]. The overhangs then undergo homology search and strand invasion catalysed by the strand exchange proteins Rad51 and meiosis-specific Dmc1 alongside various other cofactors [142, 143]. The resulting interactions are processed to form recombination products with either noncrossovers or crossovers, defined, respectively, as gene conversion or reciprocal genetic exchange between homologous chromosomes [142]. During this pairing or synapsis of dyads, a proteinaceous structure called the synaptonemal complex establishes a zipper-like connection between homologous chromosomes [144]. Reciprocal interhomolog recombination results in the formation of physical linkages between homologous chromosomes known as chiasmata, which aid coalignment of the resultant bivalents along the spindle axis for accurate segregation [145].

### 2.4. Regulation of DSBs in Meiotic Recombination

A multitude of interacting mechanisms operate in meiotic recombination to control DSB formation and resolution. This tight spatiotemporal regulation of DSB handling allows the intentional disruption of gamete DNA integrity for diversification, while ensuring coordinated cell cycling and, ultimately, generation of an intact haploid germline.

#### 2.4.1. Temporal Control of Meiotic DSBs

DSB induction is mechanistically coupled to DNA replication [146] and is subject to strict temporal regulation [147–149], occurring only between replication and chromosome segregation [150]. This ensures both coordinated chiasma formation and effective homology search for synapsis [150]. In addition, a multitude of checkpoints operate to couple DSB induction and resolution to the cell cycle to protect against the deleterious effects of unrepaired DSBs and also act to arrest or eliminate aberrant cells [151–154].

Posttranslational modifications on the Spo11-accessory protein Mer2 have been shown to contribute to the timely execution of DSB formation in meiotic recombination. Phosphorylation of Mer2 by the budding yeast cyclin-dependent kinase Cdc28 stimulates DSB formation via promotion of interactions both with itself and with other proteins involved in DSB generation including Mei4, Rec114, and Xrs2 [155]. It is known that Cdc28-Clb5 (CDK-S) activity increases during premeiotic replication and peaks before MI [156, 157] and thus may help orchestrate a concerted progression of meiotic recombination and prophase I [155]. Mer2 is also phosphorylated by Cdc7 kinase in* S. cerevisiae *to control Spo11 loading on DSB target sites [158]. It has been found that CDK-S phosphorylation on Mer2 S30 promotes DDK (Cdc7-Dbf4)-dependent phosphorylation at the adjacent S29 [157]. As well as being involved in meiotic recombination, CDK-S and DDK are both required for premeiotic S phase [156, 159] which may contribute to coordination therewith. Furthermore, it has been found that the level of DDK activity required for premeiotic S phase is lower than that supporting its postreplicative role in meiosis [159]. It has been proposed that different thresholds for both CDK-S and DDK in these processes may ensure replication precedes DSB formation [160].

Additional temporal control is afforded by meiosis-specific expression of Spo11 and accessory proteins Rec102, Rec104, Rec114, and Mei4, largely achieved via transcriptional regulation [150]. In contrast, Mer2 has been found to undergo meiosis-specific posttranscriptional regulation by a splicing factor Mer1, which itself is only expressed in meiosis [161, 162]. Although Mer2 transcription does occur in mitosis, efficient splicing by Mer1 to generate functional Mer2 is exclusive to meiosis [161].

#### 2.4.2. Spatial Control of Meiotic DSBs

Meiotic recombination and DSB formation exhibit nonrandom patterning across the genome, being more frequently localised to particular sites on chromosomes called “hotspots” [163, 164]. While exchange usually takes place between alleles, homologous recombination between nonallelic sequences with high sequence identity may occur when DSBs form in repetitive DNA sequences such as transposable elements and low-copy repeats/segmental duplications, leading to genomic rearrangements [165]. In addition, it has been shown that DSBs formed in pericentromeric regions are associated with disruption of sister chromatid cohesion leading to missegregation and aneuploidy [166]. Therefore, the location of DSBs and subsequent recombination is controlled for the maintenance of genomic integrity.

In mice, recombination hotspots have been found to be enriched for H3K4me3 histone modifications [167, 168]. These hotspots are determined by the DNA-binding histone H3K4 trimethyltransferase PRDM9, which has been found to regulate activation of recombination loci and is a key factor in specification of meiotic DSB distribution [169, 170]. Indeed, Prdm9-deficiency results in infertility and disruption of early meiotic progression in mouse models [171]. PRDM9 has been shown to directly and specifically bind a 13-mer DNA motif enriched at hotspots via its C2H2-type zinc finger array [169, 172, 173] and changes in the array affect H3K4me3 enrichment, hotspot recombination, and crossover patterns [173]. Furthermore, sequence variation in PRDM9 may underlie the intra- and interspecies differences observed in recombination hotspot distribution [169, 174]. While the exact mechanism linking hotspot specification with targeting of the DSB machinery is unclear, it has been proposed that PRDM9 may recruit Spo11, itself or via a partner protein, or may promote chromatin remodelling permissive for Spo11 binding [173].

Negative feedback systems acting both in* cis* and in* trans* also exert an inhibitory effect from one DSB on further break formation, thus limiting DSB frequency [8]. In addition, DSB formation occurs at unsynapsed chromosome segments, implicating interhomolog interactions in the control of DSB site and numbers [8, 175]. Recombination exhibits an interhomolog bias as opposed to occurring between sister chromatids [176] and it has recently been shown that total DSB number itself is a determinant of DSB repair pathway selection and development of biased recombination [177].

DSB formation is also subject to spatial regulation via control of chromosome ultrastructure. During the leptotene stage of prophase I, DSB formation occurs alongside the development of the axial element, a proteinaceous axis comprising various proteins including cohesin [145]. The sister chromatids form linear arrays of chromatin loops which are connected at their bases by the axial element, which is the precursor of the lateral element of the synaptonemal complex at pachytene [145]. Sites of DSB formation are found to map to loop sequences which are “tethered” to the axis during recombination in “tethered loop-axis complexes” [178]. ChIP-chip studies in* S. cerevisiae* have shown that Rec114, Mer2, and Mei4 stably associate with chromosome axis association sites between loops, in a manner dependent on components of the axial element [179]. Meiotic cohesin was found to control DSB formation and axial element protein and RMM recruitment at a subset of chromatin domains, while cohesin-independent DSB formation was observed in other domains [179]. Such differences in DSB machinery deposition between chromatin domains may contribute to the establishment of different recombination proficiencies, potentially providing a form of spatial regulation of DSB induction [179]. Furthermore, this interaction of RMM with axis association sites is dependent on Mer2 phosphorylation by Cdc28 which likely affords additional temporal coordination of DSB formation with the end of premeiotic replication [179].

## 3. Manipulation of DNA Topology: DSBs as Genomic Sculptors

### 3.1. Physiological DSBs in the Relief of Topological Stress

To access the information stored in compacted nuclear DNA in such processes as transcription, replication, and repair requires the repeated tangling and untangling of the DNA double helix. Such molecular contortions introduce topological entanglements into the duplex which if left unchecked could compromise genomic stability [180, 181]. Relief of such topological strain is achieved by a family of essential enzymes, the type I and type II topoisomerases. The former generate transient single-strand breaks (SSBs) while the latter family hydrolyse ATP to cut the DNA double helix and generate a transient DSB. The type II enzyme then passes one DNA duplex through the gap, and the gap is subsequently resealed [182]. Type II topoisomerase (Top II) activity in mammals is mediated by two isoenzymes, Top IIa and Top IIb, which exhibit considerable homology except in their less conserved C-termini [183, 184] and display different subcellular distributions during the cell cycle [185, 186]. Studies on Top IIa/b chimeric “tail swap” proteins and C-terminal truncations have shown that the C-terminal regions contribute to isoform-specific functionality and may modulate decatenation activity [187, 188]. Immunohistochemical studies of Top II isoform expression patterns in normal and neoplastic human tissues show that Top IIa expression is largely restricted to normal proliferative tissue and is highly expressed in certain tumour types. In contrast, Top IIb is widely expressed across both proliferative and quiescent cell populations, although at a higher level in normal proliferative tissue and tumours [189, 190].

While, in general, a Top II-induced DSB is resolved in the enzyme's catalytic cycle, it does possess a capacity for formation of abortive breaks [19] and can form longer-lived complexes in certain functions [12, 191] and both inappropriately low or excessive formation of DSB intermediates can compromise cellular viability, thus necessitating commensurate regulation [18]. The critical involvement of DNA topoisomerases in various essential cellular processes has led to the development of topoisomerase poisons for use as antibacterial [192, 193] or anticancer therapeutics [194, 195]. While catalytic inhibitors block enzymatic activity without trapping of the covalently linked DNA-enzyme intermediate cleavage complex, the majority of clinically used Top II-targeting drugs act as Top II poisons, accumulating complexes by either blocking religation or increasing complex formation. The resultant DSBs drive mitotic arrest and apoptotic cell death, illustrating the toxicity of uncontrolled DSB physiology [196]. The tight spatiotemporal regulation of Top II-mediated DSB formation enables the measured usage of these potentially deleterious DNA breaks as a physiological tool for the relief of topological stress.

#### 3.1.1. Type II Topoisomerase DSB Handling

Supercoiling, entanglement, and knotting are frequent topological obstacles encountered in DNA physiology. Type II DNA topoisomerases, through DSB induction and repair, act to resolve such obstacles using a repertoire of supercoil introduction/relaxation, catenation/decatenation, and knotting/unknotting processes [197, 198]. Eukaryotic Top II enzymes are homodimeric proteins with symmetrically paired domains, whereof each interface serves as a “gate” to control the passage of DNA strands [180]. While catalytic efficiency is a function of the structural interplay between the DNA substrate and the type II topoisomerase isoenzyme, the general mechanism is conserved across the enzyme family. The overall cycle acts to relax DNA, involving DSB induction, strand passage, and DSB repair and proceeds via a phosphotyrosine-linked DNA-enzyme intermediate [199]. The aforementioned DSB-dependent relief of DNA topological tension is of particular importance in the processes of DNA replication, transcription, and compaction into higher-order structures.

### 3.2. Physiological DSBs in DNA Replication

Replication fork arrest, such as due to impassable DNA damage, can give rise to DSBs [200, 201] which are highly mutagenic and cytotoxic and elicit a potent DDR comprising HR-mediated repair, cell cycle arrest, and possibly apoptosis, to ensure the faithful inheritance of genetic information [202–204]. Indeed, drugs which promote DSB formation or target DSB repair represent an important category of chemotherapeutics for cancer [205, 206]. In spite of all this, Top II-induced DSBs play an integral role in replication, illustrating how the careful regulation of a potentially destructive lesion can be exploited for cellular physiology.

Top II enzymes are essential in the elongation of replicating DNA chains and during segregation of newly replicated chromosomes. During semiconservative replication, the replisomal machinery at the advancing replication fork forces the intertwined DNA strands ahead of it to become overwound or positively supercoiled. These positive supercoils are redistributed behind the fork by the rotation of the replicative machinery around the helical axis of the parental duplex. This causes intertwinement of the newly replicated DNA double helices, forming precatenanes [197]. Studies in yeast reveal a necessity of either Top I or Top II in elongation, acting as replication “swivels” to relax positive supercoiling and resolve precatenanes, respectively [9, 207–209]. As replication nears completion, the unreplicated double-stranded DNA (dsDNA) segment in between the converging forks becomes too short for the action of a type I topoisomerase and replication must first be completed resulting in intertwinement of the replicated duplexes into catenated dimers [208, 210]. These intertwines are decatenated by the action of Top II to allow for chromosome separation in mitosis [211, 212]. In yeast, which only possesses a single type II topoisomerase, loss of the enzyme results in chromosome nondisjunction, accumulation of catenated dimers, and inviability at mitosis [211, 213, 214]. In contrast, it has been shown in human cells that Top IIa but not Top IIb is essential for chromosome segregation [215].

### 3.3. Physiological DSBs in Transcription

Transcription of nascent mRNA presents topological obstacles akin to those seen during replication [216]. Transcription elongation is accompanied by changes in the local supercoiled state of DNA, forming positive supercoils ahead and negative supercoils behind the transcriptional machinery [197, 198, 216, 217]. While loss of Top I or Top II alone in yeast generally does not compromise transcription,* top1 top2* temperature-sensitive double mutants exhibit significant inhibition of rRNA synthesis and, to a lesser extent, mRNA synthesis at nonpermissive temperatures [207]. In addition, double mutants accumulate negative supercoils in extrachromosomal plasmids which appear to promote transcription initiation of ribosomal minigenes on such plasmids [218, 219]. More recent studies of yeast rRNA synthesis suggest distinct roles of Top II and Top I, acting, respectively, to relieve positive supercoiling ahead of and negative supercoiling behind the advancing polymerase [11]. Pharmacological inhibition of Top II in human cells is found to decrease Pol I transcription and Top IIa depletion both impairs Pol I preinitiation complex (PIC) formation and produces a loss of DSBs at these sites. It has thus been proposed that Top IIa-induced DSBs are involved in topological changes at rDNA promoters to facilitate PIC formation [220].

As aforementioned,* top2* yeast exhibits normal levels of transcription; however it has been shown in budding yeast that loss of Top2 produces a specific reduction in Pol II-dependent transcription of genes longer than around 3 kb, attributed to defective elongation due to Pol II stalling [221]. More recently, Top II-induced DSBs have been proposed to act in a mechanism involving the transcription factor TRIM28 and DNA damage signalling to promote Pol II pause release and transcription elongation [222].

#### 3.3.1. Activity-Induced DSBs in Transcription Regulation

Transcription has long been known to stimulate HR in a locus-specific manner [223–225] and it is emerging that physiological DSBs may be involved in regulated transcription [191, 222]. Furthermore, it has recently been found that DSBs induced at transcriptionally active sites in human cells preferentially promote HR over NHEJ and this repair is dependent on the transcription elongation-associated epigenetic marker H3K36me3 [226]. It is thus becoming increasingly clear that transcription and HR-dependent DSB repair may be reciprocally regulated. Of particular interest is the emerging role of so-called “activity-induced DSBs” in transcriptional control, adding additional complexity to the integration of environmental stimuli and cell state to the modulation of transcriptional programmes. Indeed, Top IIb has previously been shown to be required for 17*β*-estradiol-dependent transactivation, via the regulated formation of site-specific longer-lived DSBs in target gene promoters [191]. Top IIb-mediated DSB formation in a target promoter has also been shown to be required for glucocorticoid receptor-dependent transactivation [227]. More recently, the spotlight has shifted to the broadening role of activity-induced DSBs in neuronal physiology and neuropathology.

#### 3.3.2. Role of Activity-Induced DSBs in Neuronal Function

Neuronal early response genes or “immediate early genes” (nIEGs) are a subset of activity-regulated genes induced with shortest latency following neuronal stimulation and typically encode transcription factors involved in transcriptional modulation for synaptic plasticity [228, 229]. Treatment of neuronal cells with etoposide, a Top II inhibitor which induces Top II-mediated DSBs [230], has been found to promote upregulation of a subset of nIEGs including* Fos*,* FosB*, and* Npas4 *[12]. This increase was not observed with other DSB-inducing agents including neocarzinostatin, bleomycin, and olaparib, suggesting a Top II-mediated mechanism [12]. Furthermore, etoposide-induced upregulation of* Fos *and* Npas4* is not affected by inhibition of DSB signalling following ATM blockade, which also suggests that nIEG induction is independent of the effects of a generic DDR to nonspecific DSBs [12]. Similar to etoposide treatment, CRISPR-Cas9-induced DSBs targeted to the* Fos* and* Npas4* promoters also produced upregulation thereof in the absence of neuronal activity. Together these indicate a role of DSBs acting within target gene promoters in the regulation of nIEG transcription [12].

Furthermore, electrical or pharmacological stimulation of cultured primary neurons revealed upregulation of* Fos* and* Npas4* mRNA accompanied by elevated *γ*H2AX levels [12], a known biomarker of DSB formation [231], suggesting the possible involvement of “activity-induced DSBs” in nIEG expression. Genome-wide ChIP-seq of *γ*H2AX following stimulation with NMDA (N-methyl-D-aspartate) demonstrated preferential clustering of yH2AX elevations at actively transcribed genes and downstream sequences. Differential peak calling on NMDA-treated samples showed that yH2AX enrichment was confined to only 21 loci including the nIEGs Fos, FosB, Npas4, Egr1, Nr4a1, and Nr4a3 among other transcription factors and noncoding RNAs, pointing to the specific localisation of DSB induction following neuronal stimulation [12]. Interestingly, *γ*H2AX was also observed to increase following pharmacological or electrical stimulation of mouse acute hippocampal slices as well as in hippocampal lysates from mice exposed to a fear-conditioning paradigm [12]. This corroborates recent evidence supporting DSB induction in mouse neurons in response to environmental stimuli in the form of spatial exploration [232]. These DSBs were found to predominate in the dentate gyrus suggesting a physiological role in spatial learning and memory [232]. Overall, these results suggest that neuronal activity specifically induces targeted DSBs involved in transcriptional regulation of nIEGs. Interestingly, studies of repair kinetics of DSBs generated in the* Fos* promoter following NMDA treatment demonstrated long-lived breaks, being repaired within 2 hours of stimulation. However, the significance of this extended lifespan is as yet unclear [12].

Other experiments showed that short hairpin RNA-mediated knockdown of* Top2b* in cultured primary neurons reduces yH2AX enrichment at nIEG exons following NMDA stimulation, pointing towards a role of Top IIb in activity-induced DSB formation at nIEGs [12]. In ChIP-seq experiments, genome-wide binding of Top IIb was found to almost quintuple following NMDA treatment and it was observed that Topo IIb enrichment clustered alongside yH2AX signals in both unstimulated and NMDA-treated cells [12]. In addition, yH2AX-enrichment following NMDA stimulation and etoposide treatment were strongly correlated, lending further support to a model of Top IIb-generated activity-induced DSBs [12]. Linking Top IIb-mediated DSB formation with activity-dependent nIEG transcription, it was found that* Top2b* knockdown in cultured primary neurons inhibits induction of nIEGs following NMDA stimulation but could be rescued by Cas9-generated DSBs targeted to the nIEG promoter [12].

While the mechanism whereby Top IIb-mediated DSBs could induce nIEG transcription is as yet unclear, one model proposed involves a functional interaction with the architectural protein CTCF [12]. CTCF organises genomic superstructure, defining chromatin architecture to control long-range interactions between different genomic loci. Major effects of CTCF action include the establishment of chromatin domains and blockade of promoter-enhancer interactions [233]. Searches for motif enrichment at Top IIb binding sites have revealed greatest clustering of CTCF under both basal conditions and NMDA treatment [12] and Top IIb itself is found to concentrate within CTCF motifs. Furthermore, Top IIb is found to coimmunoprecipitate with CTCF both under basal conditions and to a greater extent with NMDA stimulation. Genome-wide CTCF binding distribution also corresponds well with yH2AX signals at NMDA-stimulated activity-induced DSBs and following etoposide [12]. Combining these results Madabhushi et al. [12] propose a model whereby neuronal activity drives Top IIb-induced DSB formation which relieves CTCF-based topological impediments to nIEG transcription. While a recent proteomic analysis of the Top IIb proximal protein interaction network revealed interaction of Top IIb with CTCF at topologically associating domain (TAD) boundaries [234], the precise mechanism whereby activity-induced DSBs may overcome CTCF-generated obstacles is unknown. It was recently postulated that activity-induced DSBs in nIEGs lead to genomic rearrangements involving transposable elements producing a distinct form of somatic mosaicism in neurons, with implications on plasticity, network formation, and potential interplay with age-related epigenetic changes and disease [235].

Additional mechanistic complexity derives from the need for concerted repair of activity-induced DSBs. Top IIb exhibits preferential binding and cleavage at the promoters of target nIEGs, cooccupying the site with the transcriptional activator ELK1 and histone deacetylase HDAC2, the latter repressing nIEG expression [236]. Also found enriched at these sites is the tyrosyl-DNA phosphodiesterase TDP2 which acts via the NHEJ pathway to repair abortive Top IIb-induced DSBs [19, 237, 238], suggesting coordinated resolution of such physiological breaks [12]. Furthermore, TDP2-deficiency in human cells produces hypersensitivity to Top II-induced DSBs and reduces Top II-dependent transcription, illustrating the importance of regulated break repair in DSB physiology [19].

#### 3.3.3. Dysregulation of Physiological DSBs in Neuropathology

Defective DSB repair and/or loss of DSB repair components is a feature of various neuropathologies and age-associated neurodegenerative disorders, including ataxia-telangiectasia* (ATM)*, ataxia-telangiectasia-like disorder* (MRE11)*, Nijmegen breakage syndrome* (NBS1)*, and Alzheimer's disease [239–245]. In addition,* Ercc1*^Δ/-^ mice, which have compromised nucleotide excision repair, interstrand crosslink repair, and DSB repair pathways, display age-associated neurodegeneration and progressive loss of motor function [246, 247].

Increased incidence of DNA strand breakage has been identified in the brains of Alzheimer's disease (AD) patients [248, 249], and AD mouse models exhibit an elevated baseline frequency of neuronal DSBs [232]. Furthermore, amyloid-*β* (A*β*), aggregates of which are widely recognised as a key factor in AD pathogenesis, is found to increase DSB formation in primary neuronal cultures via dysregulation of synaptic transmission [232, 250]. Expression of BRCA1, an important HR DSB repair factor [251, 252], is found to be reduced in AD brains and exhibits neuronal activity-dependent regulation as well as downregulation by A*β* oligomers [253]. Moreover, short hairpin RNA knockdown of BRCA1 in wild-type mice impairs repair of activity-induced DSBs [253].

There is growing evidence suggesting that aberrant Top II activity may play a role in the aetiology of neurodevelopmental defects. Recent work involving clinical whole-exome sequencing identified a novel* TOP2B *mutation associated with intellectual disability, autistic traits, microcephaly, and developmental retardation [254] and homozygous mutations in* TDP2* have been linked with cases of intellectual disability, epilepsy, and ataxia [19]. Top IIb has been shown to be important in neuronal differentiation and survival and is upregulated during the transition from mouse embryonic stem cells to postmitotic neurons with reciprocal downregulation of Top IIa. Furthermore, loss of Top IIb in mice results in premature degeneration and apoptosis of neurons at later stages of differentiation concomitant with transcriptional deregulation of genes involved in neurogenesis and cell division [13, 14]. Pharmacological inhibition of Top I in mouse cortical neurons results in preferential downregulation of long genes, many of which are associated with autism spectrum disorder. As Top IIa/b inhibition by ICRF-193 produces a similar length-dependent transcriptional reduction, it is possible that Top II is also responsible for the regulation of certain autism-associated candidate genes [255]. Furthermore, it has been proposed that environmental and dietary sources of topoisomerase poisons and inhibitors in early neurodevelopment could be a potential cause of autism and there have been calls to factor this into food safety and risk assessments [256, 257].

Together, these findings point towards the importance of DSB repair regulation in neuronal physiology and activity-induced DSB handling, but further studies will be required to elucidate more specific roles of activity-induced DSBs in neuropathology.

### 3.4. Physiological DSBs in Chromatin Architecture

Top II is a highly abundant chromatin protein, constituting 1-2% of total chromosomal protein content in mitosis [258]. Studies of isolated chromosomes reveal association of one Top II molecule per 23,000 base pairs of DNA, largely at and around loop bases, suggesting a structural role in higher-order DNA architecture [258, 259]. Indeed, Top II binding and DSB formation sites have been identified in matrix-attachment regions,* cis-*elements which are involved in long-range chromatin organisation [260, 261]. However, photobleaching experiments have revealed both Top II isoforms to be highly mobile in the chromosomal scaffold, arguing for a more complex dynamic interaction with chromatin structure [262].

DNA topology and chromatin structural changes are known to be intricately related [263]. Early work with temperature-sensitive* top2* yeast [212] and complementation experiments with Top II-immunodepleted* Xenopus* egg extracts [264] revealed Top II to be important in chromosome condensation and segregation in mitosis, presaging the discovery of a more active role of the enzyme in chromatin remodelling. Indeed, Top II has been identified as a component of the* Drosophila* ATP-dependent chromatin remodelling complex CHRAC (chromatin-accessibility complex) [265], and mammalian BAF chromatin remodelling complexes are found to promote Top IIa activity [266]. Furthermore, Top II-mediated DNA supercoil relaxation is stimulated by the* Drosophila* protein Barren [267], which is involved in chromosome condensation and disjunction [268]. ChIP experiments in* S. pombe* demonstrate an intergenic localisation of both Top I and Top II which strongly correlates with distributions of the chromodomain ATPase Hrp1 and the histone chaperone Nap1, both involved in nucleosome disassembly [269–271]. Top II-dependent chromatin remodelling is also important in apoptotic chromosome condensation, and caspase-activated DNase (CAD) association is found to promote Top IIa activity [272]. While the exact mechanistic role of DSB handling in chromatin packing is unclear, AFM and fluorescence imaging have revealed an H1-dependent ATP-independent mechanism of Top II-mediated chromatin compaction involving clamping of DNA strands [273]. Thus, it appears that Top II DSB handling is important in highly diverse manipulations of chromatin packing and further studies are needed to elucidate the precise regulation thereof.

### 3.5. Mitochondrial DNA Maintenance and Ageing

Mitochondrial DNA (mtDNA) in humans is typically a closed circle of dsDNA [274]. As in genomic DNA, replication and transcription processes exert topological stresses on mtDNA molecules which are relieved by native topoisomerases [197, 275]. Mammalian mitochondria contain 3 topoisomerases encoded in the nucleus: Top1mt* (TOP1MT)* and Top III*α (TOP3A)* of the type I family and Top IIb* (TOP2B)* from the type II family of enzymes [275]. Although mitochondrial Top IIb is C-terminally truncated compared to its nuclear counterpart, it still retains basic type II topoisomerase DSB turnover activity, albeit with reduced processivity, and exhibits a similar pharmacological profile [276]. Top1mt deletion in mice results in elevated mtDNA transcription and induction of a stress response accompanied by considerable upregulation of Top IIb, suggesting a potential compensatory role of the type II enzyme [277]. However, both* TOP2B*^−/−^ MEFs and* TOP1MT*^−/−^*TOP2B*^−/−^ display wild-type levels of transcription and only the double-knockout elicits a stress response, suggesting nonoverlapping functions of the two molecules [277]. This picture is further complicated by the recent discovery of active full-length Top IIa and Top IIb in both murine and human mitochondria, which, although as yet uncharacterised, are likely involved in classical functions in replication and transcription like their nuclear counterparts [278].

Top IIIa is a type IA topoisomerase which is found in both the nucleus and mitochondria and has been shown to play a crucial role in the maintenance of mtDNA genomic integrity in* Drosophila*. Indeed, loss of mitochondrial import of Top IIIa in* Drosophila* is associated with a reduction in mtDNA copy number, mitochondrial dysfunction, impaired fertility, and accelerated ageing [279, 280]. The interplay between these type I and II topoisomerases in the maintenance of mtDNA and the precise roles of DSB handling therein will require further investigation.

A recently proposed but unexplored theory suggests that mitochondrial Top II may be involved in ageing [275]. With age, mtDNA undergoes attrition through entanglement and/or recombination such that mtDNA deletion is increasingly observed over time. These deletion events are found to contribute to dysfunction of the respiratory chain [281] and other tissue impairments associated with ageing [282–287]. One hypothesis postulates that age-related attrition occurs as a result of mitochondrial “poisoning” by exogenous agents, resulting in dysregulation of topoisomerase II-mediated DSB handling [275]. Many such “poisons” disrupt the ligation step of Top II, resulting in accumulation of DSB-enzyme intermediates [275]. Mammalian mitochondrial Top IIb has been found to be inhibited by the anticancer drugs amsacrine and teniposide [288] and it is possible that various known nuclear Top II poisons, ranging from the fungal toxin alternariol [289] and chemotherapeutics [290] to dietary components such as bioflavonoids from fruit and vegetables [291], may also inhibit the truncated or full-length type II topoisomerases in mitochondria. Furthermore, base oxidations, abasic sites, and other exogenously induced base modifications can act, via an unknown mechanism, to abnormally enhance Top II DSB formation [292] and promote accumulation of DNA-enzyme intermediates at sites of damage [293]. Combined with the finding that DSBs in the mitochondrial genome can induce considerable mtDNA deletion [294], Sobek and Boege speculate that exposure to mitochondrial topoisomerase “poisons” over time with age could lead to potential DSB-dependent mtDNA attrition [275]. While it is likely that mitochondrial Top II performs similar DSB-related functions to its nuclear counterpart, further studies will be needed to elucidate the precise nature of its role in mitochondria and its contribution if any to the ageing process.

Further insight into the role of Top II-induced DSBs in mitochondria may be drawn from parallels in Kinetoplastids, a group of flagellated protozoa which includes the parasitic* Trypanosoma* and* Leishmania* species, which possess a characteristic dense granule of DNA called a kinetoplast within their single mitochondrion. The kinetoplast consists of an enormous network of catenated circular mtDNA (kDNA) comprising short minicircles and much larger maxicircles and thus represents a considerable topological challenge in replication and segregation [295]. The mitochondrion contains a type II topoisomerase which localises to the kDNA [296, 297] and has been shown to be important for the maintenance of structural integrity in the kinetoplast [298], mending of holes in the kDNA following individual minicircle release for replication [299], segregation of daughter minicircles [300], and their reattachment to the kDNA network [301].

## 4. Concluding Remarks and Future Prospects

DSB physiology is thus, at its very core, a molecular surgery, reliant on both calculated incisions and timely suturing thereof, all the while, under the intense scrutiny of numerous regulatory mechanisms. While the basic biochemical mechanisms of the processes discussed above are well established, their multilateral spatiotemporal regulation remains a field of active research.

The roles of recombination defects in human disease and tumorigenesis are being increasingly appreciated and further studies will be needed to examine the nature of the dysregulation of DSB physiology in pathogenesis. New work may reveal novel pharmacological targets for human disease such as the possibility of targeting DSB signalling and repair proteins in neurodegenerative conditions such as Alzheimer's disease, to lessen aberrant DSB formation.

Novel tissue-specific roles of topoisomerases in neurophysiology are only just being uncovered with future studies required to elucidate the function of DSBs therein to understand mechanisms of learning, memory, and cognition. In addition, future work is necessary to characterise the nature of mitochondrial DSB physiology and the as yet unexplored implications on mitochondrial metabolism, disease, and ageing.

Furthermore, both topoisomerases and cancer DSB repair deficiencies are important areas of investigation in the development of anticancer therapeutics, and an understanding of the physiological roles of these targets in multiple disparate processes will inform the design of specific inhibitors with fewer off-target effects.

This account illustrates the breadth of DSB physiology across cellular biology and adds a new dimension of complexity to the regulation of genomic transactions on a basic biochemical level. The emerging role of DSB-dependent changes in topology, not only in replication and basal transcription, but also in activity-induced transcriptional changes, and potential roles in the chromatin landscape and mitochondria paint a picture of a far more active role of DSBs in fundamental DNA metabolism than previously thought.
